# Supplementation of Stingless Bee Honey from* Heterotrigona itama* Improves Antiobesity Parameters in High-Fat Diet Induced Obese Rat Model

**DOI:** 10.1155/2018/6371582

**Published:** 2018-11-21

**Authors:** Ahmad Zulkifli Mohd Rafie, Amir Syahir, Wan Amir Nizam Wan Ahmad, Mohd Zulkifli Mustafa, Abdul Razak Mariatulqabtiah

**Affiliations:** ^1^Department of Cell and Molecular Biology, Universiti Putra Malaysia, 43400 UPM Serdang, Selangor, Malaysia; ^2^Department of Biochemistry, Faculty of Biotechnology and Biomolecular Sciences, Universiti Putra Malaysia, 43400 UPM Serdang, Selangor, Malaysia; ^3^Biomedicine Science Programme, School of Health Sciences, Universiti Sains Malaysia, 16150 Kubang Kerian, Kelantan, Malaysia; ^4^Department of Neurosciences, School of Medical Sciences, Universiti Sains Malaysia, 16150 Kubang Kerian, Kelantan, Malaysia; ^5^Halal Products Research Institute, Universiti Putra Malaysia, 43400 UPM Serdang, Selangor, Malaysia

## Abstract

*Heterotrigona itama* is a common stingless bee species found in Southeast Asia. Studies on the health benefits of its honey are limited in comparison with other stingless bee species. This study examines the antiobesity benefits found in stingless bee honey (SBH) from* H. itama*. The parameters used to measure the benefits were weight change, morphological structures, and biochemical characteristics. The research was conducted by using rats that were given a high-fat diet (HFD). In total 48 male Sprague Dawley (SD) rats were given a formulated HFD to increase the levels of obesity, the HFD was administered with a value of 0.68 g/cm^2^. The duration of the treatment was six weeks, and the results show that the induction obesity using the HFD was successful. Following this, the rats were then treated with SBH (at dosages of 1000 mg/kg, 750 mg/kg or 500 mg/kg), with orlistat or with a placebo. Compared with typical obesity treatment methods, the one that used the three dosages of SBH showed a higher reduction in body mass index (BMI), percentage of body weight gain, adiposity index, and relative organ weight (ROW). The levels of liver enzymes (ALT, AST, and ALP) were also significantly lower in SBH-treated groups. The levels of triglycerides and LDL-cholesterol were significantly lower, while the level of HDL-cholesterol was significantly higher in comparison with the control obese group. In terms of morphological structures, the number of adipocyte cells was reduced, and the hepatocytes found in the liver were less prone to rupturing when treated with SBH. In conclusion, the administration of SBH led to an improvement in indicators associated with obesity reduction. SBH also possesses a hepatoprotective potential which can reduce the health risks related to obesity.

## 1. Introduction

Obesity is a condition that has become alarmingly prevalent throughout the world, and the recent increase is mainly attributed to lifestyle choices and urbanisation. Epidemic levels of obesity have led to negative impacts on overall health, including reduced life expectancy and increased risk of health-associated problems [1]. Individuals with a BMI greater than or equal to 30 are more prone to health problems including hyperlipidaemia, cardiovascular disease, high blood pressure, diabetes, stroke, arthritis, cancer, breathing problems, and metabolic syndrome [2]. The most common conditions caused by obesity are high blood pressure, diabetes mellitus, and cardiovascular diseases [3–5]. In 2015, there were approximately 20 million deaths attributed to cardiovascular diseases, accounting for 30% of all deaths worldwide. There is strong evidence to indicate many of these deaths are a direct consequence of obesity [6].

All stingless bees are from the tribe of* Meliponini*, which has many types of genera, e.g.,* Melipona*,* Trigona,* and* Heterotrigona*. Honey produced by these bee species possesses distinct aroma and taste and also has a more fluidic texture and a slower crystallization process [7]. Generally, stingless bees are found in tropical and subtropical regions of the world such as Africa, Southeast Asia, Australia, and South America. There are more than 500 species that have been identified worldwide from 32 genera, and more than 100 new species yet to be characterized [8]. Eighty-nine species from 15 genera from the Indo/Australian region have been listed [9] while, in Thailand, 32 species of stingless bee from 10 genera have been identified [8]. In Malaysia, the most common stingless bee species which are reared commercially are* Geniotrigona thoracica, Heterotrigona itama, Lepidotrigona terminata, *and* Tetragonula laeviceps *[10]. Honey produced by* G. thoracica *and* H. itama*, also known as* kelulut* honey, has shown to possess higher antioxidant activity than Manuka honey produced by western honey bees* Apis mellifera *and higher mineral contents compared to Colombian stingless bees [11]. Therefore, this type of honey is believed to boost the immune system and enhance cell function in erythrocytes associated with antimicrobial, anticancer, antiseptic, anti-inflammatory, and wound healing [11, 12]. The complex biological properties of the stingless bee honey, which consists of sugars fructose and glucose, enzymes, e.g., catalase and peroxidase, phytochemical compounds, e.g., phenolic acid and flavonoid, and other bioactive constituents such as organic acids, trace elements, amino acids (e.g. phenylalanine, alanine, tyrosine and valine), vitamins, proteins, and Maillard reaction products [10, 11, 13], might play a significant role in influencing the signalling pathway of disease development. Nonetheless, studies on the physicochemical characteristics of stingless bee honey are not detailed enough to allow the best quality practices and standards to be identified.

To expand the field of knowledge in this area, this study evaluates the effects of stingless bee honey from* H. itama* species on an obese rat model. It evaluates the effects of different dosages of stingless bee honey on weight, lipid profiles, liver function, and histopathology in each group of treated rats. These findings could pave the way for further studies that may help to produce medicinal products derived from this natural medicine, leading to the safe development of products to treat various obesity-related conditions, or as a preventive treatment for degenerative diseases such as liver disease and blood pressure.

## 2. Materials and Methods

### 2.1. Source of Honey

The multiflora stingless bee honey (SBH) was harvested from a stingless bee species of* H. Itama *(Hymenoptera: Apidae: Meliponini), which is unique Southeast Asia, found on a honeybee farm in the Kelantan district, Kelantan. The samples were stored at 4°C until they were analysed.

### 2.2. Experimental Animals

The procedures described below were approved by the Animal Ethics Committee of the Universiti Putra Malaysia (Ref: UPM/IACUC/AUP-R060/2015). A total of 48 male Sprague Dawley (SD) rats (*n*=7/group) aged three months and weighing between 200 and 250 g were used. They were acclimatized to a standard environment of 25°C with 60–70% humidity and were placed on a 12-hour light-dark cycle for one week prior to the experiment.

### 2.3. High-Fat Diet (HFD) Preparation

For every 100 g preparation, 68 g of normal rat chow in powder form was mixed with 32 g of ghee (Crispo, Malaysia) (modified from Nik Norliza et al. [14]). The HFD (32% cholesterol) was mixed with vitamin D3 (100 iu) and calcium (300 mg). The mixture was formed into marble-shaped dough samples that were kept at -40°C until solidified.

### 2.4. Obesity Induction and Animal Treatment

Control group of rats (group 1) were fed with a normal diet (ND) throughout the study. Other groups were induced with a HFD for six weeks before being assigned to different treatment groups. These included group 2: HFD with no treatment, group 3: HFD with 1000 mg/kg SBH, group 4: HFD with 750 mg/kg SBH, group 5: HFD with 500 mg/kg, and group 6: HFD with orlistat (Xenical). The body weight of each rat was recorded weekly and the changes in body weight were calculated. In the meal pattern analysis, the total quantity of food consumed by each rat was measured weekly by subtracting the remaining quantity from the quantity initially supplied. After 12 weeks, the animals were denied food for 16 hours to allow for blood collection through cardiac puncture. The retroperitoneal, epididymal, and visceral fats pads were harvested from the rat's and weighed [15].

### 2.5. Anthropometrical and Adiposity Index Determinations

The percentage of body weight, body mass index (BMI), and adiposity index was calculated using the following equations: Percentage of body weight gain = (final weight (g) - initial weight (g))/initial weight (g); BMI = body weight (g)/length^2^ (cm^2^); adiposity index (%) = ((total weight of epididymal, visceral, and retroperitoneal fat)/final body weight)) × 100.

### 2.6. Relative Organ Weight (ROW)

The relative organ weight (ROW) of liver was calculated as = (absolute organ weight (g) × 100)/final body weight of rat on sacrifice day (g).

### 2.7. Biochemical Analysis

The blood samples were centrifuged at 4000 rpm at 4°C for 15 minutes. The serum was then separated, labelled, and subjected to liver function testing (alkaline phosphatase (ALP), aspartate transaminase (AST), alanine transaminase (ALT), and serum lipid profile (total cholesterol, triglycerides, low-density lipoprotein (LDL), and high-density lipoprotein (HDL) cholesterols)). All parameters were analysed using an automated Abbott Ci8200 Biochemistry analyser which meets photometric IFCC (International Federation of Chemistry) standards.

### 2.8. Histopathological Examination (HPE) Analysis

Organs (liver and adipose tissue) were preserved in 10% buffered formalin. The tissues were fixed, processed, and embedded in paraffin wax. Then they were cut into sections and stained with haematoxylin and eosin (H&E) prior to observation under a light microscope.

### 2.9. Histological Analysis Using Scanning Electron Microscope (ScEM)

Liver tissues were dissected, removed, and fixed in McDowel-Trump Fixative at 4°C for 24 hours. Then, the fixed tissues were cut, washed, postfixed, and dehydrated. All samples were processed through critical point drying step, coated with gold and viewed under Quanta FEG 450 scanning electron microscope (ScEM) by using XTm Product Version 4.1.7.2095 viewer software.

### 2.10. Statistical Analysis

All values were expressed as mean ± standard error of mean (SEM) in each experiment. The statistical significance was determined by one-way analysis of variance (ANOVA) followed by the Bonferroni post hoc test using a GraphPad-Prism v.6.0.1 software (GraphPad, San Diego, CA). All tests were two-tailed and the significance level was set at* p*<0.05.

## 3. Results

### 3.1. The Effects of a HFD on Body Weight, BMI and Percentage of Body Weight Gain of Sprague Dawley (SD) Rats

The groups of rats fed with the HFD showed a significant increase in mean body weight, BMI, and percentage of body weight gain compared to normal control rats (group 1) (p<0.05) as shown in Table 1.

### 3.2. Effects of Stingless Bee Honey (SBH) or Orlistat Treatments on Mean Body Weight, Mean BMI, and the Percentage of Body Weight Gain of HFD Induced Obese SD Rats

Groups of obese rats treated with all dosages of SBH and orlistat showed a reduction in body weight, percentage of body weight gain, and BMI at week 6 compared to normal control rats (group 1) and untreated obese rats (group 2) (p<0.05), as shown in Table 2. Treatment using SBH at the dosage of 750 mg/kg showed the most effective result, comparable to the positive control treatment using orlistat.

### 3.3. Effects of Treatment on Adiposity Index (%) and Relative Organ Weight (ROW) of Liver

The adiposity index of control obese rats (group 2) was significantly higher compared to normal control rats (group 1) with p<0.001. The same results were seen in obese rats treated with 1000 mg/kg and 500 mg/kg of SBH. Interestingly, all treated groups of rats showed a significant reduction of adiposity index compared to control obese rats (group 2) with p<0.001. Relative organ weight (ROW) of the liver in control obese rats (group 2) reflected an increase when compared to normal control rats (Table 3). In relation to ROW, the obese rats treated with SBH at all dosages showed more desirable effects than the orlistat group or the control obese rats (group 2).

### 3.4. Effects of Treatment on Lipid Profile

The triglycerides levels in control obese rats (group 2) were significantly higher than normal control rats (group 1) (Table 4). Obese rats treated with 500 mg/kg and 750 mg/kg of SBH registered a significant difference compared to normal control rats. However, no significant difference was observed in total cholesterol level for all groups of rats. The level of LDL-cholesterol in treated obese rats (500 mg/kg, 750 mg/kg, and 1000 mg/kg of SBH) was significantly lower than the control obese rats (group 2). The HDL-cholesterol levels in control obese rats (group 2) decreased a higher rate than normal in control rats (group 1), while treated obese rats (750 mg/kg and 1000 mg/kg of SBH) presented a significant increase in the level of HDL-cholesterol compared to control obese rats (group 2) (Table 4).

### 3.5. The Effects of Treatment on Liver Enzymes

All the parameters used to measure liver enzymes in control obese rats (group 2) showed an increase when compared to normal control rats (group 1) (Table 5). All groups (500 mg/kg, 750 mg/kg, and 1000 mg/kg of SBH) showed significant decreases in the levels of AST when compared to control obese rats (group 2). For ALP and ALT levels, the obese rats treated with 750 mg/kg and 1000 mg/kg of SBH showed a significant decrease compared to control obese rats (group 2) (Table 5).

### 3.6. The Influence of Daily Dosages of Stingless Bee Honey (SBH) or Orlistat in Protecting the Liver Histology and Adipose Tissues

**Figure 1:**
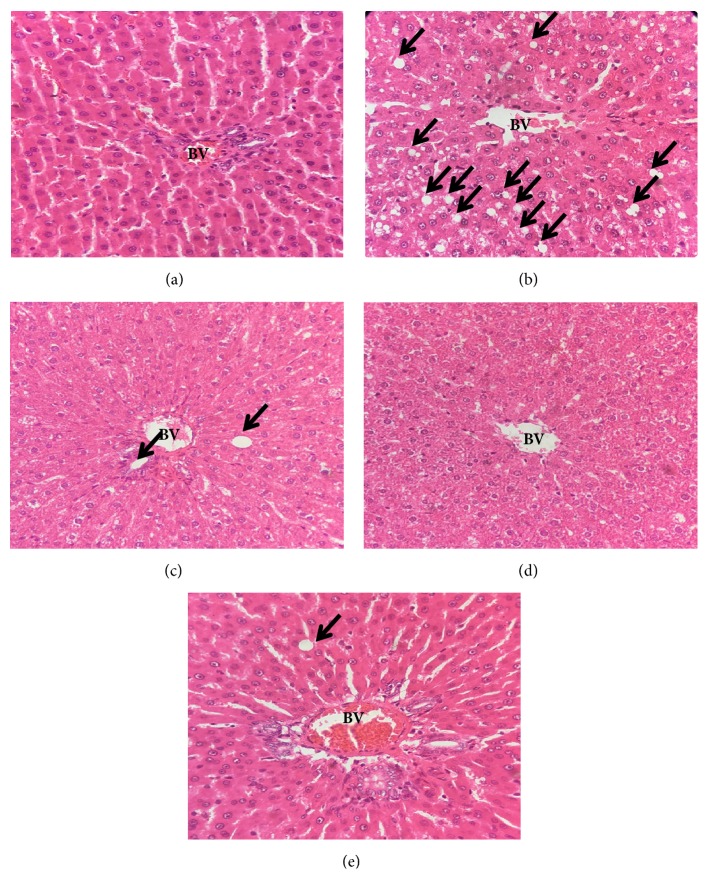
Photomicrographs show the effects of treatment and dosages of stingless bee honey (SBH) or orlistat on liver histology of obese rats, under a light microscope and following the processes of H&E with 40 x magnification. Groups: (a) normal diet rats; (b) control obese rats; (c) obese rats treated with 1000 mg/kg SBH; (d) obese rats treated with 750 mg/kg SBH; (e) obese rats treated with 500 mg/kg SBH. Black arrow (→) represents fat cells; BV: blood vessel.

Histology evaluation under a light microscope showed that livers of normal control rats (group 1) had a smooth hepatic appearance without any histological abnormalities as seen in Figure 1(a). Contrastingly, in Figure 1(b), the livers of control obese rats (group 2) showed a foamy degeneration of hepatocytes and an increased presence of bulk fat cells (+++) showing fatty changes.

**Figure 2:**
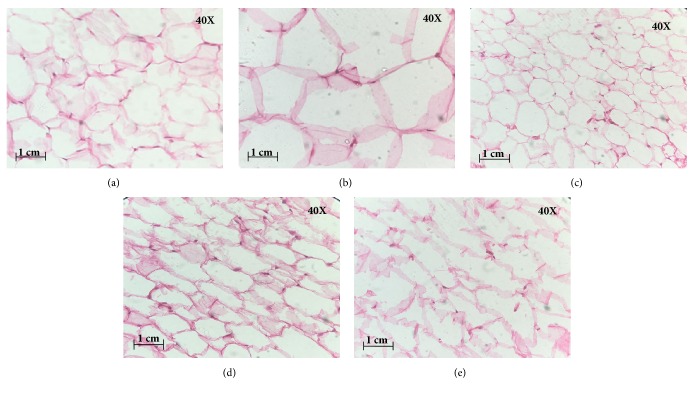
Photomicrographs show the effects of different dosages of stingless bee honey (SBH) or orlistat on visceral fatty tissue histology in obese rats, under a light microscope and after completing the processes of H&E stain with 40 x magnification. Groups: (a) normal rats; (b) control obese rats; (c) obese rats treated with 1000 mg/kg SBH; (d) obese rats treated with 750 mg/kg SBH; (e) obese rats treated with 500 mg/kg SBH.

Meanwhile, there were improvements in the liver morphology of SBH treated obese rats, where the presence of fat cells was not as high as those presented in group 2 (Figures 1(c)–1(e)). The histological images on adipose tissue of control obese rats (group 2) (Figure 2(b)) showed that adipocytes were higher than those of the normal control group (Figure 2(a)). Adipocytes in all treated groups (Figures 2(c)–2(e)) were lower than control obese rats (group 2).

### 3.7. Effects of Daily Administration of Different Treatments and Dosages of Stingless Bee Honey (SBH) or Orlistat on the Liver Using a Scanning Electron Microscope (ScEM)

**Figure 3:**
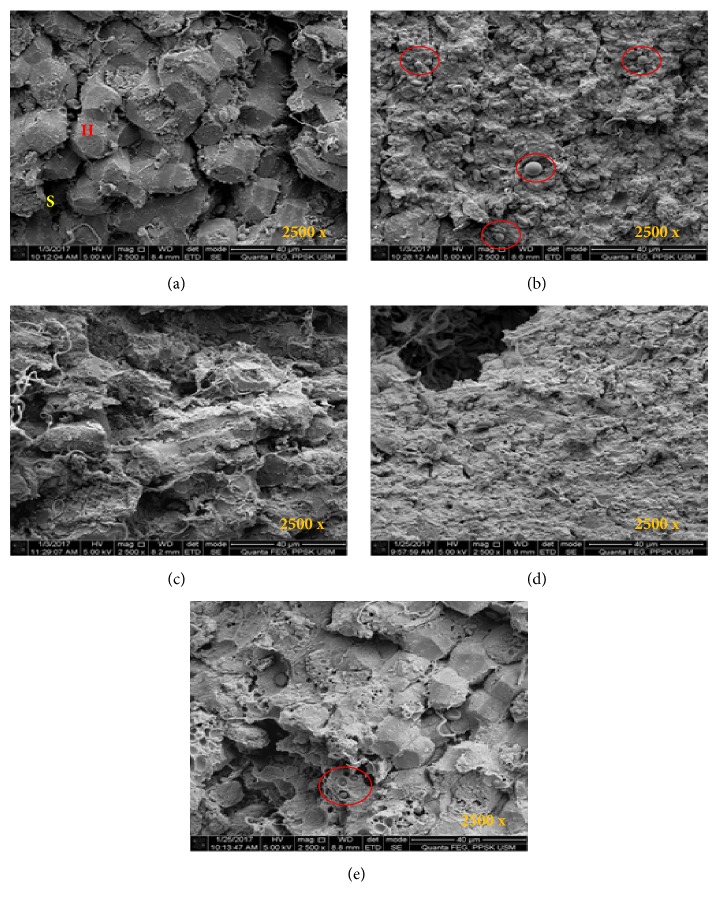
Photomicrographs show the effects of three different treatments and dosages of stingless bee honey (SBH) in liver tissue of obese rats, under a scanning electron microscope (ScEM) at 2500x magnification.** (a) **Group 1, normal architecture of liver surface (normal sinusoid and intact hepatocytes);** (b)** Group 2, obese rats' liver fed with HFD received no treatment showed presence of fat cells and have damage hepatocytes, damage sinusoid and rough surface;** (c)** Group 3, obese rats' liver fed with HFD and treated with 1000 mg/kg SBH showed fibrin formation, dilated sinusoid, and nonintact hepatocytes;** (d)** Group 4, obese rats' liver fed with HFD and treated with 750 mg/kg SBH showed smooth appearance and a few insignificant presence of fat cells;** (e)** Group 5, obese rats' liver fed with HFD and treated with 500 mg/kg SBH showed normal-like structure and a few insignificant presence of fat cells. Fat cells are indicated in red circles. H= hepatocyte; S= sinusoid.

Histology evaluation under ScEM (Figure 3) shows that the livers of normal control rats (group 1) have a normal surface, as well as a normal sinusoid and intact hepatocytes. In control obese rats (group 2), the surface of the liver displays a rough appearance with a high accumulation of fat cells. However, the surface of the liver in all treated groups (500 mg/kg, 750 mg/kg, and 1000 mg/kg of SBH) improved in terms of hepatocytes appearance and fat cell reduction.

## 4. Discussion

The consumption of a high-fat diet can lead to obesity in both humans and animals. A large accumulation of fat in the adipose tissue and a dysregulated lipid metabolism can also lead to obesity [16]. The SD rats used for this study weighed between 200 g to 220 g, and they are considered to be the most appropriate comparison for obesity studies in human [16]. The rats reached obesity by week six, as the value of BMI was more than 0.68 g/cm2, which meets the definition of obesity as set out by Novelli et al. [17]. At the end of the six-week treatment phase, the medicinal effects of SBH in relation to obesity were investigated, and the dangers of obesity were discussed. The results indicate that SBH can reduce excess weight in obese rats and that it can also help to reduce cases of liver disease and improve the lipid profile.

The initial results show that obesity was successfully induced in the rats that were fed a formulated high-fat diet. During the 6-week treatment period, the obese rats that were treated with 500 mg/kg, 750 mg/kg, and 1000 mg/kg of SBH showed a significant reduction in body weight and BMI compared to the control obese rats. These findings correlate with those given by Chepulis et al. (2009) [18] and Samat et al. (2017) [15].

The possible mechanism contributing to the reduction of body weight gain in subjects treated with honey is the insulin-mimetic effects of hydrogen peroxide that is produced from the honey and increases metabolism rates [18]. Glucose oxidase enzyme might be responsible for hydrogen peroxide production from honey through glucose oxidation [15].

Interestingly, the obese rats treated with SBH showed a reduction in the percentage of body weight gain compared to control obese rats. These results demonstrate that SBH from* H. itama* can reverse the weight gained from a high-fat diet. The findings show that the dose of 750 mg/kg is the most effective in the reduction of body weight and percentage of body weight gain. This might be because 750 mg/kg is the optimum dose for metabolic efficiency, as well as decreased fat deposition and amino acid catabolism [17].

Additionally, the adiposity index and ROW of the liver were significantly reduced in all SBH-treated groups compared to control obese rats. These findings reinforce previous data that indicates that the consumption of honey can reduce the adiposity index [19, 20]. A reduction in total body fat was evident in humans who were given honey compared to those given sucrose [21]. These studies agree with the idea that SBH reduces the total amount of fat in the body caused by the consumption of a high-fat diet.

Measurement of adiposity index and ROW is influenced by the number and size of fat cells present in body's tissues. Adipose tissue, for example, is evidently an efficient buffer against the daily flux of lipids in systemic circulation. Tissues such as skeletal muscle and liver tissue are exposed to an excess of lipids when the buffering capacity of adipose tissue is impaired [22]. Relative to adiposity index and ROW, lipid profile of serum triglycerides and LDL-cholesterol were significantly increased in obese rats in comparison with normal rats. A high level of triglycerides and LDL-cholesterol leads to plaque-like substance formation on the walls of blood vessel, blocking the natural flow of blood and increasing the chances of obesity-related diseases such as heart attack and stroke. However, all treated groups with SBH showed significant reduction in triglycerides and LDL-cholesterol. In addition, the HDL-cholesterol in all treated groups with SBH was significantly higher than that of control obese rats. Our results reinforce a study done by Yaghoobi et al. [21] which revealed that consumption of honey increased HDL-cholesterol level and reduced triglyceride and LDL-cholesterol levels in overweight subjects. This suggests that SBH is able to regulate lipid metabolism in obese rats. In this respect, SBH has demonstrated its potential as a substance that can be used for treating obese humans suffering from hypertriglyceridemia. However, no significant difference was measured in total cholesterol levels in the studied groups. This result demonstrates that the proposed formulated HFD was unable to cause hypercholesterolemia in male SD rats within a 12-week period (six weeks of induction + six weeks of treatment). This is in part due to the considerable variation among animals and humans in terms of the operation of adaptive responses to the excessive consumption of dietary cholesterol [23]. Hence, this study assumes that adaptive responses might play a role in keeping serum cholesterol levels within the normal range in many rat strains as well as in humans.

ALP, AST, and ALT are liver enzymes that serve as useful indicators in the assessment of tissue damage [24]. They are used as indicators of tissue cellular damage often caused by chemical compounds present before histological techniques can be used to detect structural damage of tissue [25]. The study reveals that ALP, AST, and ALT levels were significantly higher in control obese rats than in normal rats. This disparity demonstrates that liver problems mainly come from a HFD consumed over a long period [15]. The significant increase of ALP and AST may be due to the damage and leakage of enzymes through cellular membranes [26]. It may also be caused by the damage to the lysosomal membrane from the lysosome in the extracellular fluid [26]. Interestingly, the levels of ALP, AST, and ALT in all SBH-treated groups (500 mg/kg, 750 mg/kg, and 1000 mg/kg) are between the levels of normal rats and control obese rats. Furthermore, morphological structures of liver tissues in SBH-treated groups showed normal sinusoid, intact hepatocytes, and insignificant fat cells, which might be the consequences of the lower amount of ALP, AST, and ALT liver enzymes. By showing reduced liver enzymes and improved liver structures, it can be suggested that* H. itama* SBH possesses hepatoprotective properties and is able to reduce the risk of liver problems [27, 28].

The excessive growth of adipose tissue is associated with two growth mechanisms, hypertrophy (increased adipocyte size) and hyperplasia (increased adipocyte number) [29]. Based on the reduction of visceral adipocytes in the livers of SBH-treated groups, it is suggested that SBH may help to reduce metabolic diseases by reducing the accumulation of hepatic lipid and adipocyte hypertrophy. Adipocyte hypertrophy causes visceral obesity and is closely related to adipose tissue dysfunction, which in turn can lead to several metabolic syndromes such as type 2 diabetes, insulin resistance, dyslipidaemia, and nonalcoholic fatty liver disease [30–32]. Despite the encouraging results from this study, the exact mechanism by which SBH reduces the adipocyte size and number was not determined.

## 5. Conclusion

The results of this study revealed that, when compared to nontreated obese rats, a daily administration of SBH could significantly reduce body weight, BMI, adiposity index, and relative organ weight of obese rats giving effects comparable to that of treatment using an antiobesity drug, orlistat. The levels of triglyceride and LDL-cholesterol of all the SBH treated groups were lower, while the levels of HDL-cholesterol of all the SBH-treated groups were higher than those of the nontreated rats. This suggested that SBH was able to regulate lipid metabolism in obese rats. The levels of ALP, AST, and ALT in the liver were significantly lower in the 750 mg/kg and the 1000 mg/kg SBH-treated groups than in the nontreated obese group. This indicated the hepatoprotective abilities of* H. itama* for lowering the risk of liver related diseases. Morphological observations of the adipocytes and the hepatocytes also showed significant improvements in size and structure of the treated rats when compared to the nontreated obese rats. Consequently, it is suggested that SBH should be investigated further as it could potentially be used as a natural alternative treatment to combat obesity.

## Figures and Tables

**Table 1:** A distribution of pre- and postmean body weight, body mass index (BMI), and percentage body weight gain in each group of SD rats after they have been fed a high-fat diet for six weeks.

**Groups**	**Mean Body Weight (g)** **± SEM**		**Mean BMI (g/cm** ^**2**^ **)** **± SEM**		**Percentage of Body Weight Gain (**%**)** **Mean ± SEM**
	**Pre-induction**	**Post-induction**	**Pre-induction**	**Post-induction**	
**Group 1** **(ND)**	203.6 ± 0.8	350.6 ± 3.1	0.47 ± 0.005	0.62 ± 0.006	72.2 ± 1.1
**Group 2** **(HFD)**	201.0 ± 1.0	411.6 ± 7.7^c^	0.44 ± 0.008	0.72 ± 0.006^c^	104.8 ± 3.7^c^
**Group 3** **(HFD)**	200.6 ± 1.3	443.6 ± 8.2^c^	0.44 ± 0.008	0.72 ± 0.01^c^	121.2 ± 4.6^c^
**Group 4** **(HFD)**	196.0 ± 1.1	471.7 ± 5.1^c^	0.43 ± 0.01	0.72 ± 0.007^c^	140.7 ± 2.0^c^
**Group 5** **(HFD)**	196.4 ± 2.6	451.1 ± 3.3^c^	0.43 ± 0.011	0.71 ± 0.006^c^	130.0 ± 4.2^c^
**Group 6** **(HFD)**	196.1 ± 1.4	463.0 ± 5.5^c^	0.42 ± 0.008	0.73 ± 0.008^c^	136.1 ± 2.7^c^

Results are presented as means ± SEM, *n *= 7.

Values are statistically significant at *p* < 0.05.

Note: **a**: *p<0.05* when compared to ND;** b**: *p<0.01* when compared to ND; **c**: *p<0.001* when compared to ND.

ND: normal diet; HFD: high-fat diet.

**Table 2:** A distribution of pre- and post-mean body weight, mean BMI, and percentage of body weight gain in each study group of induced-obese SD rats after daily administration of stingless bee honey (SBH) for a period of six weeks.

**Groups**	**Mean Body Weight (g) ± SEM**		**Mean BMI ± SEM**		**Percentage of Body Weight Gain (**%**) ± SEM**
	**Pre-intervention**	**Post-intervention**	**Pre-intervention**	**Post-intervention**	
**Group 1** **(ND)**	350.6 ± 3.1	468.1 ± 3.6	0.62 ± 0.006	0.73 ± 0.009	33.58 ± 1.2
**Group 2** **(Obese rats; HFD)**	411.6 ± 7.7^a^	549.9 ± 11.7	0.72 ± 0.006	0.83 ± 0.013	33.73 ± 2.8
**Group 3** **(Obese rats; HFD+1000 mg/kg SBH)**	443.6 ± 8.2^a^	497.3 ± 19.2^b^	0.72 ± 0.010	0.73 ± 0.019^b^	11.98 ± 3.1^a, b^
**Group 4** **(Obese rats; HFD+750 mg/kg SBH)**	471.7 ± 5.1^a^	460.3 ± 13.6^b^	0.72 ± 0.007	0.69 ± 0.016^a, b^	-2.30 ± 2.9^a, b^
**Group 5** **(Obese rats; HFD+500 mg/kg SBH)**	453.6 ± 2.6^a^	504.3 ± 21.6^b^	0.71 ± 0.006	0.73 ± 0.018^b^	11.14 ± 4.6^a, b^
**Group 6** **(Obese rats; HFD+ orlistat)**	463.0 ± 5.5^a^	405.9 ± 6.6^a, b^	0.73 ± 0.008	0.65 ± 0.009^a, b^	-12.36 ± 0.6^a, b^

Results are presented as means ± SEM, *n* = 7.

Values are statistically significant at *p* < 0.05.

Note: ^**a**^significantly different compared to normal control rats (group 1); ^**b**^significantly different compared to control obese rats (group 2).

ND: normal diet; HFD: high-fat diet; SBH: stingless bee honey.

**Table 3:** Adiposity index (%) and relative organ weight (ROW) of liver in SD rats after daily administration of stingless bee honey (SBH) for a period of six weeks.

**Parameters**	**Groups**					
	**Group 1 (ND)**	**Group 2** **(Obese rats; HFD)**	**Group 3** **(Obese rats; HFD+1000 mg/kg SBH)**	**Group 4** **(Obese rats; HFD+750 mg/kg SBH)**	**Group 5** **(Obese rats; HFD+500 mg/kg SBH)**	**Group 6** **(Obese rats; HFD+ orlistat)**
**Adiposity Index (**%**)**	3.8 ± 0.2	10.1 ± 0.5^a^	5.6 ± 0.3^b^	4.6 ± 0.5^b^	6.8 ± 0.8^a, b^	3.7 ± 0.3^b^
**Relative Organ Weight (Liver)**	3.5 ± 0.1	9.4 ± 0.4^a^	3.0 ± 0.1^b^	2.7 ± 0.2^b^	3.4 ± 0.1^b^	3.2 ± 0.1^b^

Results are presented as means ± SEM, *n* = 7.

Values are statistically significant at *p* < 0.05.

Note: ^**a**^significantly different compared to normal control rats (group 1); ^**b**^significantly different compared to control obese rats (group 2).

ND: normal diet; HFD: high-fat diet; SBH: stingless bee honey.

**Table 4:** Levels of triglyceride, total cholesterol, LDL-cholesterol, and HDL-cholesterol in SD rats after daily administration of stingless bee honey (SBH) for a period of six weeks.

**Parameters**	**Groups**					
	**Group 1 (ND)**	**Group 2** **(Obese rats; HFD)**	**Group 3** ** (Obese rats; HFD+1000 mg/kg SBH)**	**Group 4 (Obese rats; HFD+750 mg/kg SBH)**	**Group 5** ** (Obese rats; HFD+500 mg/kg SBH)**	**Group 6** **(Obese rats; HFD+ orlistat)**
**Triglyceride (mmol/L)**	0.35 ± 0.02	1.33 ± 0.22^a^	0.73 ± 0.08^b^	0.83 ± 0.08^a, b^	0.78 ± 0.08^a, b^	0.50 ± 0.05^b^
**Total cholesterol (mmol/L)**	1.93 ± 0.11	1.93 ± 0.19	1.47 ± 0.14	1.80 ± 0.03	1.90 ± 0.15	1.75 ± 0.06
**LDL-cholesterol (mmol/L)**	0.18 ± 0.03	0.70 ± 0.13^a^	0.17 ± 0.06^b^	0.12 ± 0.03^b^	0.17 ± 0.02^b^	0.53 ± 0.15^a^
**HDL-cholesterol (mmol/L)**	1.37 ± 0.10	0.93 ± 0.11^a^	1.33 ± 0.10^b^	1.35 ± 0.09^b^	1.30 ± 0.09^b^	1.17 ± 0.07

Results are presented as means ± SEM, *n* = 7.

Values are statistically significant at *p* < 0.05.

Note: ^**a**^significantly different compared to normal control rats (group 1); ^**b**^significantly different compared to control obese rats (group 2).

ND: normal diet; HFD: high-fat diet; SBH: stingless bee honey.

**Table 5:** The effects of the stingless bee honey treatment on levels of liver enzymes in SD rats after daily administration of stingless bee honey (SBH) for a period of six weeks.

**Parameters**	**Groups**					
	**Group 1** **(ND)**	**Group 2** **(Obese rats; HFD)**	**Group 3** **(Obese rats; HFD+1000 mg/kg SBH)**	**Group 4** **(Obese rats; HFD+750 mg/kg SBH)**	**Group 5** **(Obese rats; HFD+500 mg/kg SBH)**	**Group 6** **(Obese rats; HFD+ orlistat)**
**Alkaline Phosphatase (U/L)**	89.8 ± 4.8	187.3 ± 26.9^a^	104.2 ± 25.2^b^	122.0 ± 9.3^b^	140.5 ± 9.0	128.3 ± 20.9
**Aspartate Aminotransferase (U/L)**	195.8 ± 14.3	389.7 ± 45.7^a^	154.5 ± 19.0^b^	183.33 ± 18.0^b^	206.3 ± 18.9^b^	334.5 ± 23.2^a^
**Alanine Aminotransferase (U/L)**	55.0 ± 5.7	118.8 ± 32.7^a^	51.0 ± 4.9^b^	56.3 ± 5.6^b^	64.8 ± 8.6	53.3 ± 2.1^b^

Results are presented as means ± SEM, *n* = 7.

Values are statistically significant at *p* < 0.05.

Note: ^**a**^significantly different compared to normal control rats (group 1). ^**b**^Significantly different compared to control obese rats (group 2).

ND: normal diet; HFD: high-fat diet; SBH: stingless bee honey.

## Data Availability

The data used to support the findings of this study are available from the corresponding author upon request.

## Abbreviations

ALP: Alkaline phosphatase
ALT: Alanine transaminase
AST: Aspartate transaminase
BMI: Body mass index
HDL: High-density lipoprotein
HFD: High-fat diet
LDL: Low-density lipoprotein
ND: Normal diet
ROW: Relative organ weight
SBH: Stingless bee honey
SD: Sprague Dawley
ScEM: Scanning electron microscope
SEM: Standard error of mean.
